# Stage Management at a Hospital

**Published:** 1911-03-25

**Authors:** 


### STAGE MANAGEMENT AT A HOSPITAL.

(By A HOSPITAL SECRETARY.)

The man in the street whose inclination or calling has not brought him in personal touch with a big London hospital can have little or no practical notion of the amount of actual stage management that is necessary if the institution is to be kept before the eyes of the public in a way that is likely to draw financial help.

One reads from time to time informing little paragraphs in relation to the work of a particular hospital. Some observant newspaper readers may have remarked that certain hospitals appear to secure much more advertisement and notoriety of the right sort than do other institutions. It may be thought that this fact is due to reasons derived from the daily occurrences at the hospitals in question. But such is not of necessity the case. It would be perfectly fair to say that all hospitals of any size or standing have an equal chance to add to their reputation and advertise themselves. A certain big hospital, for instance, obtains constant and clever advertisement, and the majority of interested persons, no doubt, suppose that this is because it is one of the largest institutions in the country, and because it is frequently visited by members of the Royal Family and other distinguished sympathisers. If the truth dawned, however, it would transpire that there is somewhere or other a nimble brain at work patiently hatching out paragraphs which give items of news and interest.

The casualty room in any general hospital furnishes ample "copy," if only those in authority were to realise it. It may be urged that it is not right and proper for a hospital to draw attention in print to the painful or the gratifying circumstances which happen week after week. Yet there are many ways of doing a thing, and if a thing is done with taste and with subtlety, it pays to get the name of the hospital before the public as often as possible. The name should be repeated and repeated until it sinks into the minds of the public.

The stage management of a big hospital is not worth carrying out unless it be for financial reasons, for it entails a great deal of work. Of course, it goes without saying that the internal arrangements of a modern hospital are as well-nigh perfect as human organisation can make them; if they are not, then the hospital suffers in countless ways. That work is a domestic duty; it cannot rightly be termed stage management.

Nowadays no voluntary hospital can afford to ignore the arts of advertisement, and, therefore, no prosperous hospital is conducted on loose or inadequate lines. Stage management is the atmosphere that appeals to the eye and the brain of those who are likely to be of help, or whom it is necessary to please with a view to favours to come. It does not matter in what way one looks at the question; there would be no stage management if it were not with a view to winning the appreciation of the spectator; there would be no stage management if it were not in the sure and certain hope that the efforts would bring benefit in some way or other, or, at any rate, cause valuable comment and advertisement. Needless to say, it is quite possible to do the wrong thing in stage management; indeed, no other art is so full of pitfalls. The true stage manager must have imagination and insight; he must know exactly what effect certain little pictures, whether conveyed in print or scenic effects, will have upon the minds of those before whom they are brought. In these days a hospital chairman, or a hospital secretary, who has not such gifts would be better employed in a position where few fresh ideas and little resource are necessary.

Some hospitals gain the reputation of being "lucky" or successful, while others are recognised as "unfortunate." There may be mitigating circumstances, but if the real cause could be ascertained it would be found to rest with those who are responsible for the stage management of the place.

Some of my readers will recollect reading in the early months of last year that the cuckoo had been heard in the grounds of a certain suburban hospital. It will also be recollected that very late in the same year an attractive and droll little note appeared in the papers to the effect that roses were still blooming in the grounds of the same hospital. Such harmless and pleasant little paragraphs could not fail to impress the name of the institution upon one, and doubtless thousands of newspaper readers noted the news with smiling satisfaction. It is not easy to get sub-editors to "pass" these pleasantries, but there are ways and ways. After all, the chief way is to make the paragraphs fresh rather than futile, snappy rather than stale, and pointed rather than pointless. Some hospitals when visited by a member of the Royal Family will carelessly dispose of the news in a three-line paragraph, while other better-served hospitals when similarly honoured by a visit from the highest in the land are able to secure half a column in half a hundred different publications.

"Work is as it is done," and work when it is thoughtful and judicious always tells. A man with a little hammer can get through a brick wall if he has the will and the means of purchasing further hammers when the first one is worn out, yet another man with a little hammer would be filled with dismay were he asked to make a hole in a stout wooden partition.

It would pay some of the institutions of the metropolis (where there are so many) if they were in these days of eager thirst for information to employ a special publicity agent. Newspaper readers are always eager for items of news which are served up in a really new, attractive, and cheerful way. Hospitals have a personal interest for over two-thirds of mankind in civilised countries, and it is for this reason that news concerning the centres of sickness should be freely supplied. The Board of Management of a big institution might well make it one of their first duties to see that the light of their work is not hidden under a bushel.

